# Novel Insights in the Potential of Halogenated Polyketide–Peptide Molecules as Lead Compounds in Cancer Drug Discovery

**DOI:** 10.3390/ijms24076208

**Published:** 2023-03-25

**Authors:** Valentina Pellicioni, Germana Esposito, Giulia Greco, Ivan Cruz-Chamorro, Fabio Ferrini, Piero Sestili, Roberta Teta, Carmela Fimognari, Valeria Costantino

**Affiliations:** 1 Dipartimento di Scienze per la Qualità della Vita, Alma Mater Studiorum-Università di Bologna, 47921 Rimini, Italy; valentina.pellicion2@unibo.it; 2 “TheBlueChemistryLab”, Dipartimento di Farmacia, Università di Napoli Federico II, 80131 Napoli, Italy; germana.esposito@unina.it (G.E.); roberta.teta@unina.it (R.T.); valeria.costantino@unina.it (V.C.); 3 Dipartimento di Chimica “Giacomo Ciamician”, Alma Mater Studiorum-Università di Bologna, 40126 Bologna, Italy; giulia.greco9@unibo.it; 4 Departamento de Bioquímica Médica y Biología Molecular e Inmunología, Facultad de Medicina, Universidad de Sevilla, 41009 Seville, Spain; icruz-ibis@us.es; 5 Instituto de Biomedicina de Sevilla, IBiS/Hospital Universitario Virgen del Rocío/CSIC/Universidad de Sevilla, 41013 Seville, Spain; 6 Dipartimento di Scienze Biomolecolari, Università di Urbino Carlo Bo, 61029 Urbino, Italy; f.ferrini2@campus.uniurb.it (F.F.); piero.sestili@uniurb.it (P.S.)

**Keywords:** *Smenospongia aurea*, natural products, polyketide, molecular networking, LC-MS, PCD pathways, apoptosis, ferroptosis, lipid peroxidation, smenolactone D

## Abstract

In this interdisciplinary study, we selected two compounds, namely, smenamide A, a peptide–polyketide, and smenolactone D, a polyketide, as models because they are representative of two different classes of molecules isolated from the marine sponge *Smenospongia aurea*. The organic extract of *Smenospongia aurea* was analyzed using a combination of high-resolution LC-MS/MS and molecular networking, a recently developed method for automated LC-MS data analysis. The analyses were targeted to highlight clusters made by chlorinated compounds present in the extracts. Then, the two model compounds were analyzed for their bioactivity. Data reported here show that smenamide A did not exhibit a cytotoxic effect, while smenolactone D was cytotoxic on different tumor cell lines and was able to induce different types of cell death, including ferroptosis and apoptosis.

## 1. Introduction

Natural compounds represent an interesting source of bioactive molecules and play an important role in the drug discovery process, especially in the oncology field [1]. Indeed, among all the newly discovered anticancer drugs, about 60% are derived from natural compounds [2].

The earth’s surface is covered by 70% of oceans and seas, which represent the natural habitat of about 80% of the plant and animal species living in the entire world [3,4]. For this reason, over the past 60 years, researchers have focused on the marine habitat to exploit its enormous potential [2,5]. The modern technological advances have facilitated the access to marine biota and have permitted the discovery of a plethora of new bioactive compounds [6]. However, the supply of marine-derived compounds remains an expensive and arduous process, thus chemical synthesis or semi-synthesis processes are often employed.

Sponges are multicellular porous organisms belonging to the phylum Porifera that can live in all marine areas, from poles to the equator [7]. Among all marine organisms, they represent the most thriving reservoir of bioactive compounds. This is partly due to the enormous diversity of their intraphylum. Indeed, in the last 10 years, 2400 new molecules were isolated from more than 600 species of sponges [8]. Since sponges have no immune system nor physic defense mechanisms, they produce secondary metabolites as chemical defenses [9]. Chemically, those metabolites can be classified as nucleosides, terpenoids, polyketides, sterols, alkaloids, macrolides, sugars, steroids, shikimic acid derivatives, peroxides, terpenoids, fatty acids, amino acid derivatives, and peptides [2,10,11,12]. Several compounds isolated from marine sponges have been found to exhibit anticancer activity ascribed to their ability to interfere with several cellular and molecular events and to induce apoptosis in cancer cells [2,10].

Apoptosis is the programmed cell death (PCD) mechanism mostly involved in the activity of numerous clinically employed anticancer drugs [13]. However, therapeutic approaches based exclusively on apoptosis induction are characterized by several issues, mainly due to the development of cancer cells’ resistance to apoptosis [13]. Recently, it has been shown that there are non-apoptotic, or non-canonical, PCD mechanisms, such as ferroptosis and necroptosis. These cell death pathways can be activated along with apoptosis or when it is inhibited [14]. Therefore, since pro-apoptotic strategies may lead to therapeutic failure, the identification of compounds capable of inducing non-canonical cell death could constitute an alternative or complementary therapeutic approach.

Sponges represent the most important source of marine natural bioactive compounds [15,16,17,18]. Several compounds isolated from sponges have been found to induce pro-apoptotic effects [10,19,20,21]. Furthermore, it was recently reported that a limited number of sponge-derived molecules, such as heteronemin, fascaplysin, and mycalols, triggers non-canonical PCD mechanisms, either singly or in combination with apoptosis, in hepatocellular carcinoma cells or in non-small cell lung cancer cells [22,23,24].

The Caribbean sponge *Smenospongia aurea* (*S. aurea*) is a case-study marine organism. In fact, a novel class of secondary metabolites has been isolated starting from the first paper published in 2013 reporting the isolation of smenamide A. Since this first report, 10 novel molecules have been isolated and their bioactivity has been investigated [25,26,27,28]. Moreover, the total synthesis of two stereoisomers of smenamide A, namely, ent-smenamide A and 16-epi-smenamide A [29], were reported and the 16-epi-analogue of smenamide A and eight simplified analogues in the 16-epi series were prepared using a flexible synthetic route [30]. In the present study, we analyzed the extract of *S. aurea* using a combination of high-resolution LC-MS/MS and molecular networking, a recently developed method for automated LC-MS data analysis. The analyses were targeted to highlight clusters made by chlorinated compounds present in the extracts. Then, we further investigated the cytotoxic potential of smenamide A (**1**) and smenolactone D (**2**), a hybrid peptide/polyketide and a polyketide [25,27], respectively. In particular, we investigated their ability to induce canonical and non-canonical cell death mechanisms.

## 2. Results and Discussion

**Figure 1:**
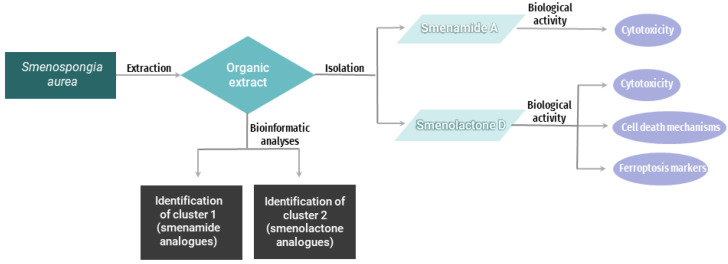
Single flow diagram representing the overall methodology adopted.

The reported results are derived from an interdisciplinary study combining chemical, bioinformatic, and biological analyses, as schematically illustrated in Figure 1.

### 2.1. The Bioinformatic Analyses Highlight the Presence of Two Clusters of Novel Chlorinated Molecules in S. aurea Extract

A specimen of *S. aurea* collected along the coast of Little Inagua (Bahamas Islands) was extracted as previously described [27]. Sponge organic extracts were combined and analyzed for chlorinated metabolites by LC-HRMS and LC-HRMS/MS on an LTQ Orbitrap instrument. The raw analytical data were pre-processed using the mzMine program [31] and were studied using molecular networking, a bioinformatic tool that allows to easily evaluate and capture multiple datasets in one visual network, overcoming the problem of dealing with a huge amount of data that are difficult to manage. In particular, the mgf MS2 data file generated by mzMine was submitted to the online platform at the Global Natural Products Social Molecular Networking website (gnps.ucsd.edu) [32] and the network was visualized using the Cytoscape program [33].

**Figure 2:**
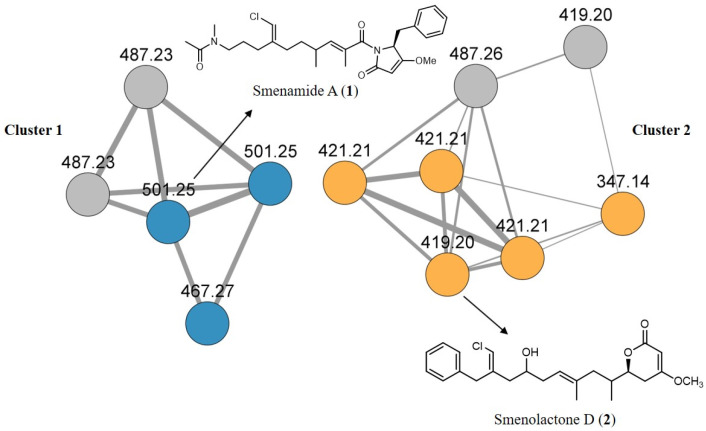
Molecular network obtained from the LC-MS/MS analyses of extracts from *S. aurea*. Edge thickness is relative to the cosine score. Cluster 1 represents smenamide analogues (in blue are known compounds and in light grey are unknown compounds), cluster 2 represents smenolactone analogues (in orange are known compounds and in light grey are unknown compounds).

The molecular network analysis led to the identification of two main clusters: cluster 1 containing smenamide analogues and cluster 2 containing smenolactone analogues. In detail, cluster 1 contained smenamides A and B, along with smenamide C and two minor unidentified isomeric compounds at m/z 487.23, previously detected and described in reference [34], while cluster 2 contained smenolactone D, smenolactone A at m/z 347.14, three isomeric compounds at m/z 421.21 corresponding to smenolactone B, smenolactone C, and trichophycin B, and two unidentified analogues at m/z 487.26 and m/z 419.20 (Figure 2).

The bioinformatic analyses allowed the fast dereplication of the MS-MS data of the sponge extract and highlighted the presence of two clusters of novel chlorinated molecules in a very first stage, avoiding long and time-consuming chromatographic purifications in this initial phase. The analysis revealed that there are four unknown analogues (colored in grey in the visual representation) that will be the objects of further studies.

To deeper investigate the cytotoxic potential of these classes of molecules on tumor cells, one compound for each class was selected and the cytotoxic mechanism was studied. Because smenamide A (**1**) and smenolactone D (**2**) were isolated in a greater amount from the organic extract of *S. aurea*, they were selected for the cytotoxicity studies.

### 2.2. Smenolactone D Is Cytotoxic in a Panel of Human Cancer Cell Lines

To assess the cytotoxic potential of the marine-derived compounds, we tested smenamide A (**1**) and smenolactone D (**2**) on a panel of human cell lines, representing hematological and solid tumors: lymphoblastic T cells (Jurkat), estrogen- and progesterone-receptor positive breast adenocarcinoma cells (MCF-7), and epidermoid carcinoma cells (A-431).

**Figure 3:**
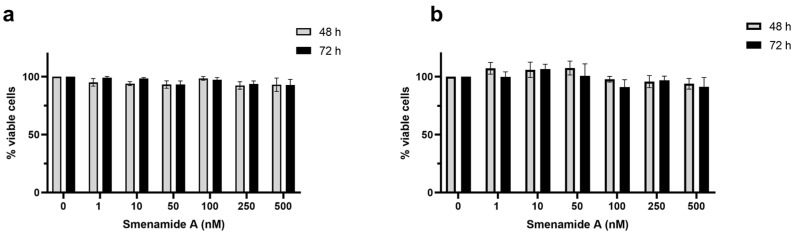
Smenamide A is not cytotoxic in all tested cell lines. Percentage (%) of viable A-431 (**a**) and MCF-7 (**b**) cells after treatment with increasing concentrations of smenamide A for 48 or 72 h.

Smenamide A was tested at nanomolar concentrations (1–500 nM) on MCF-7 and A-431 cells. After 48 and 72 h of treatment, it elicited no cytotoxic effect in either cell line (Figure 3). Similar results were also recorded on Jurkat cells, where smenamide A concentrations were increased up to 20 μM. Despite the difference of an order of magnitude, the decrease in cell viability induced by the compound was not statistically different from untreated cultures. These results differ from those observed by Teta et al. on human Calu-1 non-small-cell lung carcinoma cells, where the compound induced cytotoxicity at nanomolar concentrations, achieving an IC_50_ (half-maximal cytotoxic concentration) of 48 nM [25]. This discrepancy suggests that the cytotoxic activity of smenamide A strongly depends on the type of the tested cancer cell line.

**Figure 4:**
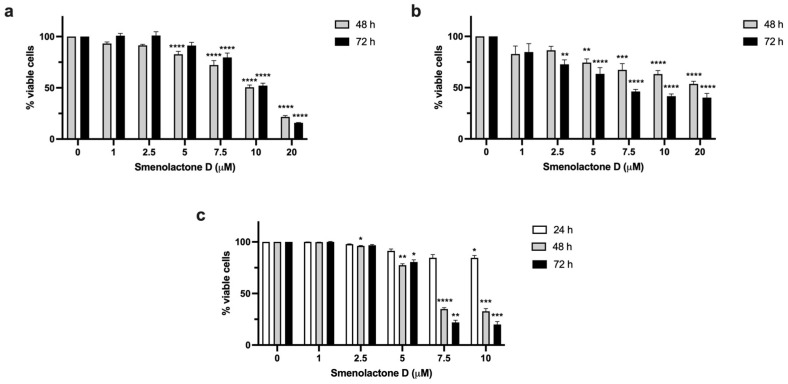
Smenolactone D is cytotoxic in all tested cell lines in a time- or concentration-dependent manner. Percentage (%) of viable A-431 (**a**) MCF-7 (**b**) and Jurkat (**c**) cells after treatment with increasing concentrations of smenolactone D for 48 or 72 h. * *p* < 0.05, ** *p* < 0.01, *** *p* < 0.001; **** *p* < 0.0001 compared to untreated cells.

Smenolactone D (**2**) reduced the cell viability of all the tested cell lines in a dose-dependent manner (Figure 4), with Jurkat cells being the most sensitive, as demonstrated by the IC_50_ values (Table 1). In A-431-treated cells, the percentage of cell viability after 48 and 72 h was very similar (Table 1). These results indicate that the cytotoxic activity of smenolactone D reaches a plateau. On the other hand, the cytotoxic effect on MCF-7 and Jurkat cells was time-dependent, as shown by the lower IC_50_ value following 72 h of treatment compared to that obtained after 48 h (Table 1). Our findings are consistent with those previously observed in the work of Teta et al., where smenolactone D (**2**) inhibited the proliferation of MCF-7 cells (concentration 2 µM) and of pancreatic cancer cells BXPC-3 (concentration 1 µM) [27].

Jurkat cells exhibited the lowest IC_50_ values (Table 1). Thus, the following experiments were performed on this cell line treated with smenolactone D at 7.5 µM for 48 h.

### 2.3. Smenolactone D Induces Apoptosis and Ferroptosis in Jurkat Cells

**Figure 5:**
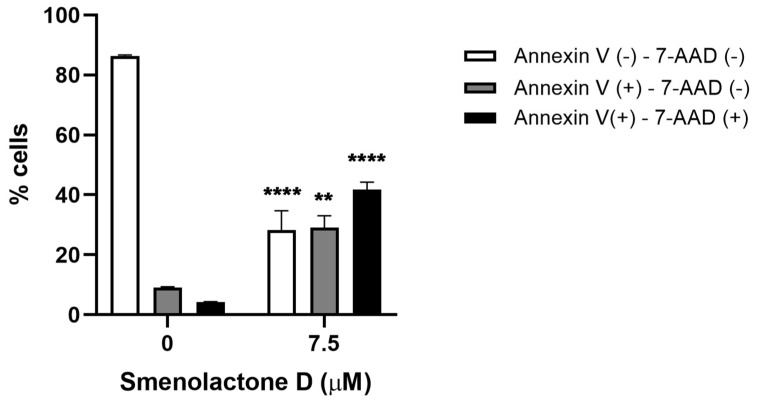
Smenolactone D induces PCD in Jurkat cells. Percentage (%) of viable cells (annexin-V^−^/7-AAD^−^), in the early stages of PCD (annexin-V^+^/7-AAD^−^) or necrotic/in the late stages of PCD (annexin-V^+^/7-AAD^+^) following 48 h treatment with smenolactone D (7.5 µM). ** *p* < 0.01; **** *p* < 0.0001 compared to untreated cells.

To characterize the type of cell death responsible for the cytotoxic activity of smenolactone D (**2**), Jurkat cells were analyzed by flow cytometry after the staining with Guava Nexin Reagent containing annexin-V and 7-amino-actinomycin D (7-AAD). This assay allows to determine whether the induced cell death mechanism is programmed or not. Indeed, annexin-V binds to phosphatidylserine (PS), which is present in the outer side of the cell membrane after its translocation from the inner side, an event occurring in the early stages of many PCDs, such as apoptosis or necroptosis [35]. However, in cells with disrupted cell membranes, as necrotic or cells in the late stages of PCD, PS can be bound even if it is located on the inner side of cell membranes. For this reason, the cell-impermeant fluorescent dye 7-AAD was employed as a marker of membrane integrity. Hence, the combination of annexin-V/7-AAD allows the discrimination of three cellular populations: viable cells (annexin-V^−^/7-AAD^−^), cells in early stages of PCD (annexin-V^+^/7-AAD^−^), and necrotic cells or cells in late stages of PCD (annexin-V^+^/7-AAD^+^). Treatment of Jurkat cells with smenolactone D resulted in a significant increase in the fraction of annexin-V^+^/7-AAD^+^ cells (41.76% in treated cells compared to 4.25% in untreated cells), and an increase in annexin-V^+^/7-AAD^−^ cells (29.04% in treated cells compared to 9.10% in untreated cells) (Figure 5). These results indicate that treatment with smenolactone D (**2**) led a smaller, but significant fraction of cells to PCD, while a larger fraction was necrotic or in the late stages of PCD.

**Figure 6:**
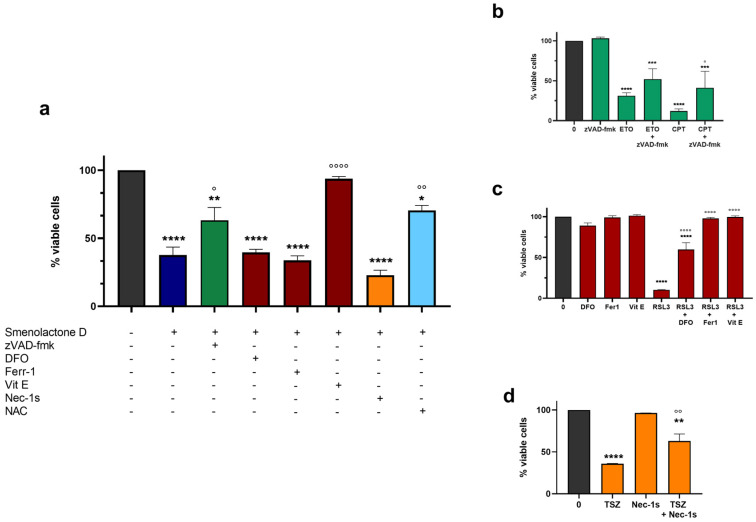
Smenolactone D triggers apoptosis and ferroptosis. (**a**) Percentage (%) of viable cells following 1 h pre-treatment with different pharmacological inhibitors and subsequent 48 h treatment with smenolactone D (7.5 µM). * *p* < 0.05, ** *p* < 0.01; **** *p* < 0.0001 compared to untreated cells. ° *p* < 0.05, °° *p* < 0.01; °°°° *p* < 0.0001 compared to cells treated with smenolactone D 7.5 µM. (**b**) Percentage (%) of viable cells following 1 h pre-treatment with pan-caspase inhibitor zVAD-fmk and subsequent 48 h treatment with ETO (1 µM) or CPT (2.5 µM) used as positive controls. *** *p* < 0.001; **** *p* < 0.0001 compared to untreated cells. ° *p* < 0.05 compared to cells treated with CPT. (**c**) Percentage (%) of viable cells following 1 h pre-treatment with different pharmacological ferroptosis inhibitors, such as DFO (10 µM), Ferr-1 (1 µM), and Vit E (100 µM) and subsequent 48 h treatment with ferroptosis positive control RSL3 (0.3 µM). **** *p* < 0.0001 compared to untreated cells. °°°° *p* < 0.0001 compared to cells treated with RSL3. (**d**) Percentage (%) of viable cells following 1 h pre-treatment with necroptosis inhibitor Nec-1s and subsequent 48 h treatment with TSZ (TNFα 75 ng/mL, SM164 500 nM, and zVAD-fmk 50 µM). ** *p* < 0.01; **** *p* < 0.0001 compared to untreated cells. °° *p* < 0.01 compared to cells treated with TSZ. Green bars: apoptosis, red bars: ferroptosis, orange bars: necroptosis, light blue bar: ROS inhibitors or positive controls, black bars: untreated cultures, dark blue bar: smenolactone D 7.5 µM.

To characterize the cell death pathway triggered by smenolactone D (**2**), specific pharmacological inhibitors of different types of PCDs were used. Specifically, cells were pre-treated with (i) the pan-caspase inhibitor carbobenzoxy-valyl-alanyl-aspartyl-[O-methyl]-fluoromethylketone (zVAD-fmk), (ii) the iron chelator deferoxamine mesylate (DFO), the lipid ROS (reactive oxygen species) scavenger vitamin E (Vit E) and ferrostatin-1 (Ferr-1), and (iii) the RIP-1 (Receptor Interacting Protein 1) kinase activity inhibitor necrostatin-1s (Nec-1s) to determine the involvement of apoptosis, ferroptosis, or necroptosis, respectively. In addition, NAC (N-acetyl-L-cysteine), a potent ROS scavenger and GSH (glutathione) precursor, was used to assess their involvement in smenolactone D-induced cytotoxicity. As positive controls, etoposide (ETO) and camptothecin (CPT) were used for apoptosis, RAS-selective lethal 3 (RSL3) was used for ferroptosis, and tumor necrosis factor α (TNFα) + SM164 + zVAD-fmk (TSZ) was used for necroptosis. Pre-treatment with Vit E induced an almost complete recovery of viability of cells treated with smenolactone D (**2**) (97.3% versus 37.7% of smenolactone D-treated cells), indicating that its cytotoxic activity is strongly dependent on lipid peroxidation (Figure 6). Pre-treatment with NAC also significantly counteracted smenolactone D-induced cell death (70.5% versus 37.7%), indicating that GSH depletion and ROS accumulation represent key events for its cytotoxicity (Figure 6). Of note, pre-treatment with zVAD-fmk antagonized the cytotoxic activity of 2 (63.1% versus 37.7%), underlying the involvement of the apoptotic cell death process in the cytotoxicity of smenolactone D (Figure 6). In contrast, pre-treatment with the other two ferroptosis inhibitors, DFO and Ferr-1, and the necroptosis inhibitor Nec-1s, did not rescue the Jurkat cells’ viability. On the whole, these results led us to hypothesize that smenolactone D (**2**) may induce multiple PCD mechanisms, specifically apoptosis and ferroptosis.

The involvement of caspase-dependent apoptosis in the cytotoxicity of smenolactone D (**2**) is unequivocal, as demonstrated by the recovered Jurkat cells’ viability after the inhibition of caspase activity. On the other hand, since two out of three ferroptosis inhibitors failed to protect Jurkat cells from the cytotoxic effect of smenolactone D, it was necessary to better understand the involvement of the ferroptotic cell death mechanism in the cytotoxic activity of smenolactone D (**2**). Ferroptosis is activated by an iron-dependent accumulation of lipid ROS, in particular lipid hydroperoxides in cell membranes, resulting from the direct or indirect inhibition of GPX4 [36,37]. Lipid peroxidation can occur by two processes: (i) autoxidation, which is an iron-catalyzed spontaneous peroxyl chain reaction mediated by radicals, (ii) a process catalyzed by several non-heme iron-dependent enzymes. Among them, only lipoxygenases (LOXs) have been associated with ferroptotic cell death [38]. LOX inhibitors can suppress enzymatic lipid peroxidation, while lipid autoxidation can be blocked by radical-trapping antioxidants (RTA) [39]. Although both Ferr-1 and Vit E have been identified as RTAs, their different mechanisms of action may explain their different effects on compound **2**-induced ferroptosis inhibition. Indeed, Vit E (α-tocopherol) has been shown to inhibit ferroptosis through the inhibition of LOXs, specifically 15-LOX [40], which is a recognized key regulator of the ferroptotic process [39], while Fer-1 is a poor inhibitor of 15-LOX but an excellent inhibitor of autoxidation [41].

**Figure 7:**
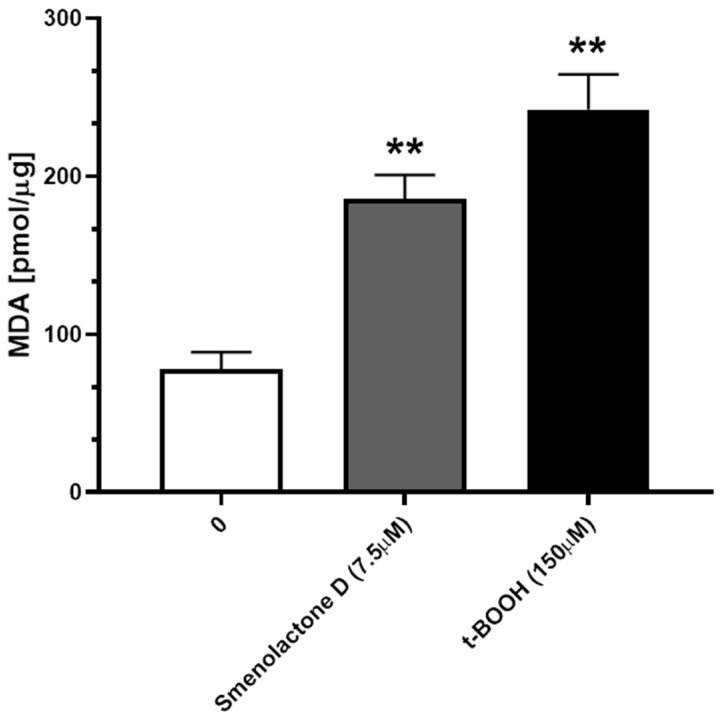
Smenolactone D induces lipid peroxidation. Levels of MDA on Jurkat cells treated with smenolactone D 7.5 µM for 48 h. t-BOOH 150 µM was used as positive control. ** *p* < 0.01 compared to untreated cells.

To further confirm the involvement of lipid peroxidation in smenolactone D-induced cytotoxicity, we analyzed the cellular levels of malondialdehyde (MDA), one of the final products of lipid peroxidation. Treatment with smenolactone D 7.5 µM for 48 h resulted in the accumulation of fairly high levels of MDA in the Jurkat cells (Figure 7), an effect that has been observed in parallel Jurkat cells’ samples exposed for 1 h to 150 µM of tert-butyl hydroperoxide (t-BOOH), a prototypic organic hydroperoxide employed as a positive control.

**Figure 8:**
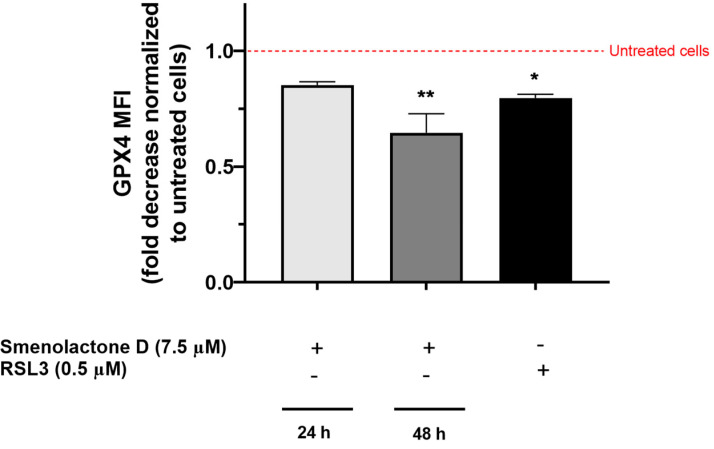
Smenolactone D induces a time-dependent decrease in GPX4 protein expression. GPX4 protein expression is indicated as MFI fold decrease compared to untreated cells. Jurkat cells were treated with smenolactone D (7.5 µM) for 24 or 48 h, or with RSL3 (0.5 µM) used as positive control for 24 h. * *p* < 0.05; ** *p* < 0.01 compared to untreated cells.

Ultimately, to prove the link between lipid peroxidation and ferroptosis, we evaluated GPX4 protein expression, whose depletion causes the accumulation of lipid ROS [37]. GPX4 is the only member of the GPX protein family able to convert phospholipid hydroperoxides to phospholipid alcohols, using GSH as cofactor [42]. The inhibition of GPX4 can occur through two main mechanisms: (i) direct inhibition, such as that induced by the progenitor of GPX4 inhibitors RSL3, which inactivates GPX4 by binding to its active site selenocysteine [43], (ii) indirect inhibition, such as that induced by erastin, which blocks the Xc- glutamate/cysteine antiport system and GSH biosynthesis, leading to reduced levels of intracellular cysteine, an essential substrate for GSH synthesis [43,44]. Treatment with smenolactone D (**2**) for 24 or 48 h time-dependently decreased GPX4 expression (Figure 8). In particular, after 24 h, the mean fluorescence intensity (MFI) was reduced by 14.7%, slightly less than that recorded with RSL3 at the same treatment time (20.3%), while after 48 h, GPX4 MFI was further decreased by 35.3%. Although further experiments are needed to characterize whether smenolactone D induces ferroptosis in a direct or indirect manner, our results demonstrate that this cell death mechanism is involved in its cytotoxic activity.

## 3. Materials and Methods

### 3.1. Isolation and Structural Elucidation

For this study, smenamide A (**1**) and smenolactone D (**2**) were obtained following our standard procedure as reported in [25,27]. Briefly, a sample of *S. aurea* collected by scuba along the southwest coast of Little Inagua (Bahamas Islands) was extracted with MeOH (4 × 4 L), MeOH, and CHCl_3_ in different ratios (2:1, 1:1, 1:2) and then with CHCl_3_ (2 × 4 L). The MeOH extract was partitioned between H_2_O and BuOH; the BuOH layer was combined with the CHCl_3_ extracts and concentrated in vacuo. The resulting organic extract was subjected to reversed-phase chromatography using a column packed with RP-18 silica gel. The fraction eluted with MeOH/H_2_O (9:1) was partitioned in a two-phase system composed by H_2_O (160 mL), MeOH (260 mL), CHCl_3_ (140 mL), and AcOH (5 mL). The organic layer, containing smenamides and smenolactones, was subjected to two subsequent reversed-phase HPLC separation affording pure compound 1 (smenamide A, Pubchem CID: 137628576) and 2 (smenolactone D, Pubchem CID: not available) (all data are available in Supplementary Materials SI). An aliquot of 1.0 mg of each compound was used for cytotoxic assays [45].

Structural elucidation of two compounds was achieved through a combination of HR-LCMS spectrometry and mono and bidimensional NMR spectroscopy (Tables S1 and S2 and Figures S1–S5 in Supplementary Materials).

### 3.2. MZmine and Molecular Networking

LC-HRMS and LC-HRMS/MS experiments were performed using a Thermo LTQ Orbitrap XL high-resolution ESI mass spectrometer coupled with an Agilent model 1100 LC system, which included a solvent reservoir, an inline degasser, a binary pump, and a refrigerated autosampler. LC-HRMS/MS raw files were directly imported into MZmine 2.23 to deconvolute chromatographic peaks, distinguish between isomeric compounds based on their retention times, and remove isotope and adduct peaks, as described in detail in our previous paper [27]. Then, a molecular network [32] was generated submitting the mgf MS2 data files from MZmine in the online workflow at GNPS (https://gnps.ucsd.edu/). The parent mass tolerance and MS/MS fragment ion tolerance were both set at 0.02 Da. In the resulting molecular network, edges were refined to have a cosine score above 0.6 and more than six matched peaks. The spectra in the network were then compared with those in GNPS spectral libraries. To observe a matching between network spectra and library spectra, a cosine score above 0.7 and at least six matched peaks were required. Once the basic molecular network was generated, it was visualized with Cytoscape 3.2.122 [33].

### 3.3. Cell Culture and Treatment

Human acute T leukemia (Jurkat), human epidermoid carcinoma (A-431), and estrogen and progesterone receptor-positive human breast cancer (MCF-7) cells were purchased from LGC Standard (LGC Group, Middlesex, UK). Jurkat and A-431 cells were propagated in Roswell Park Memorial Institute (RPMI) 1640 medium containing 10% heat-inactivated fetal bovine serum (FBS), 1% L-glutamine 200 mM, and 1% penicillin (10,000 units)/streptomycin (10 mg/mL) solution (all provided by Euroclone, Pero, Italy). MCF-7 cells were propagated in Eagle’s Minimal Essential medium (EMEM, Euroclone) supplemented with 10% FBS, 1% L-glutamine 200 mM, 1% penicillin/streptomycin, and 0.1% insulin. All cell lines were maintained at 37 °C under 5% CO_2_ in a humidified incubator.

All compounds were dissolved in dimethyl sulfoxide at a final concentration of 10 mM. Cells were treated with increasing concentrations of each compound for 24, 48, or 72 h.

To characterize the mechanisms of cell death elicited by smenolactone D, Jurkat cells were pre-treated for 1 h with different inhibitors and then exposed to smenolactone D (7.5 μM) for 48 h. The following pharmacological inhibitors were used: the pan-caspase inhibitor zVAD-fmk (BioVision, Waltham, MA, USA) at 50 μM to inhibit apoptosis; the inhibitor of kinase activity of RIP-1 Nec-1s (Merck, St. Luis, MO, USA) at 75 μM to inhibit necroptosis; the inhibitor of ROS generation and lipid peroxidation Ferr-1 (Merck) at 1 μM; the iron chelator DFO (Acros Organics, Thermofisher Scientific, Geel, Belgium) at 10 μM and the peroxyl radical scavenger Vit E (Merck) at 100 μM to inhibit ferroptosis. NAC (Merck) at 10 mM was used as the ROS scavenger. Different positive controls were used: ETO (1 μM) and CPT (2.5 μM) to induce apoptosis, RSL3 (0.3 μM) to induce ferroptosis, and TNFα (75 ng/mL), SM164 (500 nM), and zVAD-fmk (50 µM) for necroptosis induction.

### 3.4. Cell Viability Assays

Jurkat cell viability was analyzed by flow cytometry using the cell-impermeable DNA-intercalating fluorescent dye Sytox^TM^ Green (Thermo Fisher Scientific, Waltham, MA, USA). Briefly, Jurkat-treated cells were resuspended in phosphate buffer saline (PBS) 1X containing SYTOX^TM^ Green 10 nM and incubated for 20 min in the dark at room temperature. Fluorescence was measured with the Guava EasyCyte 6-2L flow cytometer (Guava Technologies, Merck Millipore, Darmstadt, Germany).

Cell viability of A-431- and MCF-7-treated cells was analyzed spectrophotometrically using the reagent 4-methylumbelliferyl heptanoate (MUH, Merck), which is hydrolyzed by viable cells generating a fluorescent molecule. Briefly, after treatment, A-431 and MCF-7 cells were washed in PBS 1X and incubated in PBS 1X containing MUH 0.01 mg/mL for 30 min at 37 °C and 5% CO_2_. Victor X3 microplate reader (Perkin Elmer, Waltham, MA, USA) was used to measure fluorescence (330 nm excitation; 450 nm emission).

For all cell lines, cell viability was expressed as a percentage (%) of living cells, obtained from the normalization of the fluorescence of treated samples to untreated cells.

### 3.5. Annexin-V Assay

Guava Nexin Reagent (Luminex, Austin, TX, USA) was used to discriminate PCD events from necrotic events, as it contains 7-AAD and phycoerythrin (PE)-labeled annexin-V. Briefly, Jurkat cells were treated with smenolactone D (7.5 μM) and after 48 h cells were diluted in 100 μL of Guava Nexin Reagent. After 20 min in the dark at room temperature, cells were analyzed by flow cytometry, as previously reported [46].

### 3.6. Determination of Lipid Peroxidation 

Lipid peroxidation was assessed in cell lysates with a colorimetric kit (Sigma Aldrich, St. Louis, MO, USA), as described in [47]. The level of MDA in cell’s supernatants was measured at 532 nM using a spectrophotometer. 

### 3.7. Evaluation of GPX4 Expression

Jurkat cells treated with smenolactone D (7.5 μM) for 24 or 48 h were collected, fixed with 4% formaldehyde, and permeabilized with 90% cold methanol. Then, cells were washed in wash buffer, containing PBS 1X with 1% bovine serum albumin, and incubated with anti-GPX4 primary antibody (1:150, Invitrogen, Thermo Fisher Scientific) for 1 h at 4 °C. After washing, cells were stained with the anti-rabbit secondary antibody (1:200; Thermo Fisher Scientific). After 1 h at 4 °C, cells were washed and analyzed by flow cytometry. GPX4 protein expression was expressed as fold change of the MFI recorded in treated cells to that of untreated samples. Cells treated with RSL3 0.5 μM for 24 h were used as the positive control.

### 3.8. Statistical Analysis

All results were expressed as the mean ± SEM of at least 3 independent experiments. Statistical analyses were performed using one- or two-way ANOVA and Tukey or Bonferroni as post-tests. IC_50_ values (concentrations that inhibit 50% of cell viability) were calculated from a dose–response curve using the non-linear regression [log(inhibitor) versus normalized response]. The statistical software GraphPad InStat 8.0 version (GraphPad Prism, San Diego, CA, USA) was used, and *p* < 0.05 was considered significant.

## 4. Conclusions

The enormous potentiality of marine organisms to synthesize molecules by their secondary metabolism pathways can be used to generate lead compounds in drug-discovery studies. Thus, we performed a study on the cytotoxic potential of two molecules, i.e., smenamide A (**1**) and smenolactone D (**2**), isolated from samples of the marine sponge *S. aurea.* Due to the number of new molecules isolated from its organic extracts, *S. aurea* represents a well-known case-study marine sponge. The two compounds have been chosen as the model because they are representative of two different classes of chlorinated molecules isolated from this marine sponge. Smenamide A is a hybrid peptide–polyketide molecule containing a dolapyrrolidinone moiety, which is produced through a mix-biogenesis pathway in which the peptide part is conjugated with a lipophilic chain. Smenolactone D is a polyketide, which shares with smenamide A the characteristic chlorovinyl moiety.

Overall, our results demonstrated an interesting in vitro cytotoxic activity of smenolactone D, while smenamide A was not active on either hematological or solid tumor cell models. Smenolactone D was cytotoxic on all tested tumor cell lines, even if at different concentration. It is worth noting that its activity is dependent on the activation of two PCD pathways: apoptosis and ferroptosis. Smenolactone D elicited lipid peroxidation through the downregulation of GPX4 protein expression. In light of the issues associated with the development of resistance to anticancer therapy based on apoptosis induction, this multifaceted mechanism is a favorable aspect. For this reason, smenolactone D can be considered a promising lead compound in disease management such as cancer and can pave the way for the convenient synthesis of improved analogues. However, it is worth drawing attention to the fact that no data are available on the effects of smenolactone D on non-transformed cells. Its selectivity for cancer cells should be explored to better understand its pharmacotoxicological potential.

## Figures and Tables

**Table 1:** IC_50_ values calculated from dose–response curves of Jurkat, A-431, and MCF-7 cells treated with smenolactone D for 24, 48, or 72 h.

Cell Line	IC_50_ (µM)		
	24 h	48 h	72 h
Jurkat	37. 43	7.08	6.39
A-431	n.c.	10.68	10.76
MCF-7	n.c.	26.58	8.32

n.c.: not calculated.

## Data Availability

All data in this study can be requested from the corresponding author (carmela.fimognari@unibo.it, C.F.).

## Supplementary Materials

The following supporting information can be downloaded at: https://www.mdpi.com/article/10.3390/ijms24076208/s1.
