# Supplementary material for: Novel Insights in the Potential of Halogenated Polyketide–Peptide Molecules as Lead Compounds in Cancer Drug Discovery
Source: Int J Mol Sci. 2023 Mar 25;24(7):6208. doi: 10.3390/ijms24076208 (PMC10094022; doi:10.3390/ijms24076208)
Supplement: Supplementary file 1 [file ijms-24-06208-s001.zip › ijms-2304026-supplementary.pdf]

International Journal of
Molecular Sciences

MDPI

Supplementary material

Novel Insights in the Potential of Halogenated Polyketide–Peptide Molecules as Lead Compounds in Cancer Drug Discovery

Table of Contents

Figure S1. Smenamide A (**1**)

TableS1. NMR data for smenamide A (**1**) (700 MHz, CD_3OD)

Figure S2. Positive ion mode high-resolution ESI MS spectrum of smenamide A (**1**)

Figure S3. Positive ion mode high-resolution ESI MS/MS spectrum of smenamide A (**1**)

Figure S4. Smenolactone D (**2**)

Table S2. NMR data for smenolactone D (**2**) (700 MHz, CD_3OD)

Figure S5. Positive ion mode high-resolution ESI MS/MS spectrum of smenolactone D (**2**)

International Journal of
Molecular Sciences

MDPI

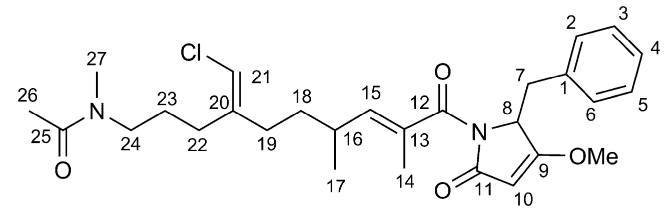

1

Figure S1. Smenamide A (1): Colorless amorphous solid, HRESIMS (positive ion mode, MeOH) *m/z* 523.2326 ($[M + Na]^+$, $C_{28}H_{37}ClN_2O_4Na^+$, calcd. 532.2334), *m/z* 501.2509 ($[M + Na]^+$, $C_{28}H_{38}ClN_2O_4^+$, calcd. 501.2515); MS isotope pattern: M (100%), M + 1 (32%, calcd. 31.5%), M + 2 (37%, calcd. 36.0%), M + 3 (10%, calcd. 10.6%,); HRESIMS/MS (parent ion *m/z* 523.23, $C_{28}H_{37}ClN_2O_4Na^+$): *m/z* 487.2557 ($C_{28}H_{36}N_2O_4Na^+$, calcd. 487.2567), 397.2092 ($C_{21}H_{30}N_2O_4Na^+$, calcd. 397.2098), 320.1384 ($C_{16}H_{24}ClNO_2Na^+$, calcd. 320.1388), 284.1618 ($C_{16}H_{23}NO_2Na^+$, calcd. 284.1621), 244.0941 ($C_{12}H_{15}NO_3Na^+$, calcd. 244.0944), 226.0836 ($C_{12}H_{13}NO_2Na^+$, calcd. 226.0838), 202.0472 ($C_9H_9NO_3Na^+$, calcd. 226.0475); ^1H and ^{13}C NMR: Table S1; UV (MeOH): $\lambda_{max}(\varepsilon)$ 287 nm (8200), 246 nm (46000), 225 nm (92000); CD (MeOH): $\lambda_{max}(\Delta\varepsilon)$ 238 (+33), 219 (−30).

Position		***Z*-Conformer**		***E*-Conformer**			
		**δ_H [Mult., *J* (Hz)]**	**δ_C [Mult.]**	**δ_H [Mult., *J* (Hz)]**	**δ_C [Mult.]**	**COSY**	**HMBC**
1		–	135.6 (C)	–	135.6 (C)		
2/6		6.99 (m)	130.8 (CH)	6.99 (m)	130.8 (CH)	3/5	4
3/5		7.23 (ovl)	129.4 (CH)	7.23 (ovl)	129.4 (CH)	2/6	1
4		7.23 (ovl)	128.3 (CH)	7.23 (ovl)	128.3 (CH)	2/6	
7	a	3.37 (ovl)	34.8 (CH_2)	3.37 (ovl)	34.8 (CH_2)	7b, 8	1, 2/6, 8, 9
	b	3.19 (m)		3.19 (m)		7a, 8	2/6
8		5.02 (ovl)	60.5 (CH)	5.02 (ovl)	60.5 (CH)	7a, 7b	
9		–	179.5 (C)	–	179.5 (C)		
10		5.04 (br. s)	95.5 (CH)	5.02 (br. s)	95.5 (CH)		8, 11
11		–	170.7 (C)	–	170.7 (C)		
12		–	172.3 (C)	–	172.2 (C)		
13		–	132.1 (C)	–	132.1 (C)		
14		1.77 (d, 1.5)	13.7 (CH_3)	1.78 (d, 1.5)	13.7 (CH_3)	15	12, 13, 15
15		5.36 (br. d, 10.2)	144.1 (CH)	5.36 (br. d, 10.2)	144.1 (CH)	14, 16	
16		2.45 (m)	33.4 (CH)	2.48 (m)	33.4 (CH)	15, 17, 18a	
17		0.98 (d, 6.5)	20.4 (CH_3)	1.00 (d, 6.5)	20.6 (CH_3)	16	15, 18, 19
18	a	1.51 (ovl)	36.1 (CH_2)	1.52 (ovl)	35.9 (CH_2)	16, 19a, 19b	19
	b	1.28 (ovl)		1.30 (ovl)		19a, 19b	
19	a	2.19 (ovl)	33.2 (CH_2)	2.23 (ovl)	33.2 (CH_2)	18a, 18b, 19b, 21	20, 21
	b	2.06 (ovl)		2.05 (ovl)		18a, 18b, 19a, 21	20
20		–	143.1 (C)	–	142.8 (C)		
21		5.93 (br. s)	113.9 (CH)	5.97 (br. s)	114.1 (CH)	19a, 19b	20, 22
22	a	2.22 (m)	28.1 (CH_2)	2.26 (m)	28.0 (CH_2)	22b, 23	20, 21
	b	2.15 (m)		2.18 (m)		22a, 23	20
23		1.64 (m)	25.9 (CH_2)	1.70 (m)	26.6 (CH_2)	22a, 22b, 24	
24		3.36 (ovl)	48.6 (CH_2)	3.33 (ovl)	51.5 (CH_2)	23	22, 23, 25, 27
25		–	172.9 (C)	–	172.7 (C)		

International Journal of
Molecular Sciences

26	2.08 (s)	21.7 (CH_3)	2.07 (s)	21.1 (CH_3)	27	25
27	3.03 (s)	36.6 (CH_3)	2.88 (s)	33.7 (CH_3)	26	24, 25
OMe	3.97 (s)	59.7 (CH_3)	3.97 (s)	59.7 (CH_3)		9

Table S1. NMR data for smenamide A (1) (700 MHz, CD3OD).

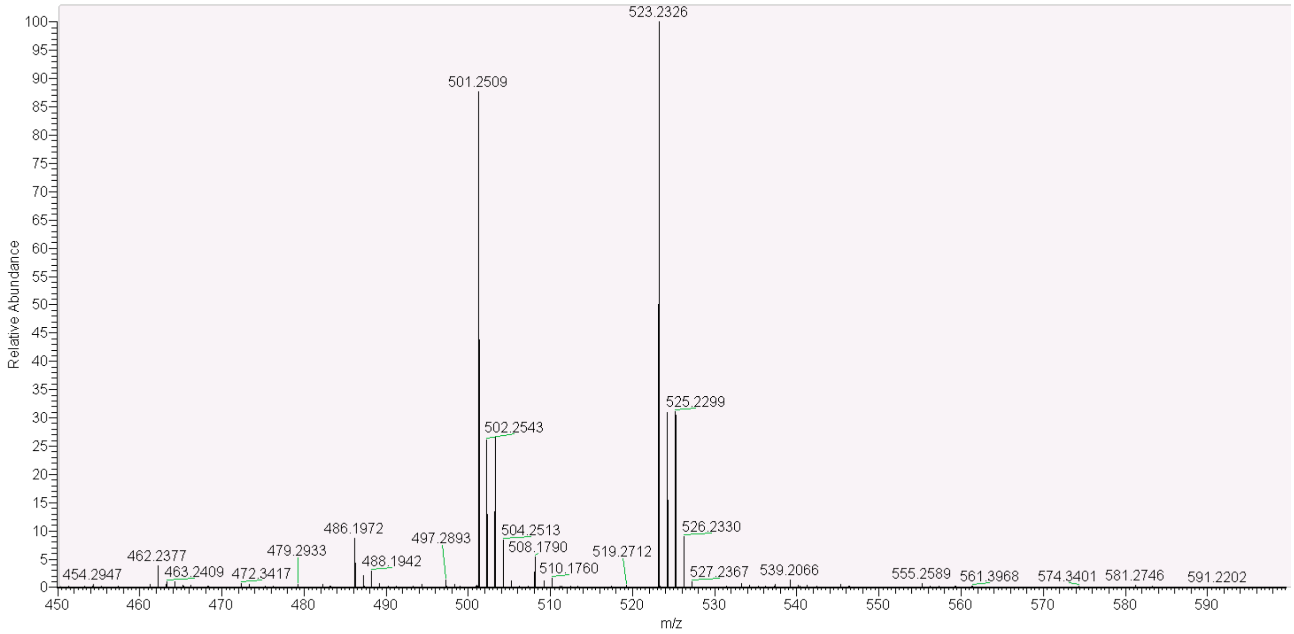

Figure S2. Positive ion mode high-resolution ESI MS spectrum of smenamide A (**1**).

The positive ion mode high-resolution ESI mass spectrum of smenamide A (**1**) displayed $[M + H]^+$ and $[M + Na]^+$ pseudomolecular ion peak at *m/z* 501.2508 and 523.2326, respectively. Both ions showed intense (32%) M + 2 isotope peaks, suggesting the presence of one atom of chlorine, and were indicative of the molecular formula $C_{28}H_{37}ClN_2O_4$ (calcd. 501.2515 for $C_{28}H_{38}ClN_2O_4$ and 523.2334 for $C_{28}H_{37}ClN_2O_4Na$). The peak at *m/z* 487.2557 ($[M - HCl + Na]^+$) in the HRMS/MS spectrum confirmed the presence of chlorine in the molecule.

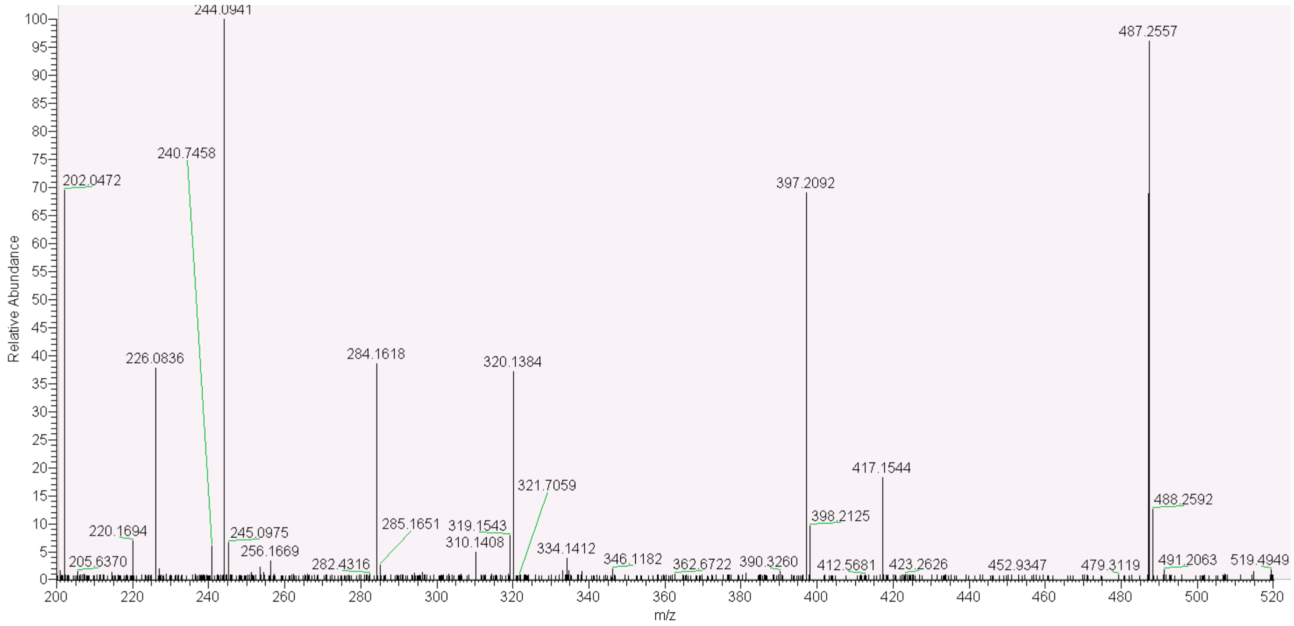

Figure S3. Positive ion mode high-resolution ESI MS/MS spectrum of smenamide A (**1**), parent ion at *m/z* 523.23.

International Journal of Molecular Sciences

MDPI

Optical rotations were measured using a Jasco P-200 polarimeter. UV spectra were measured using a Beckman Coulter DU-800 spectrophotometer. ECD spectra were recorded on a Jasco J-715 spectrophotometer using a 1-mm cell. NMR spectra were determined on Varian Unity Inova spectrometers at 700 MHz; chemical shifts were referenced to the residual solvent signal (CD_3OD: δ_H 3.31, δ_C 49.00).
High-resolution ESI-MS spectra were performed on a Thermo LTQ Orbitrap XL mass spectrometer. The spectra were recorded by infusion into the ESI source using MeOH as the solvent.
High performance liquid chromatography (HPLC) separations were achieved on a Varian Prostar 210 apparatus equipped with a Varian Prostar 325 UV-Vis detector.
The organic layer obtained from acid partition was subjected to reversed-phase HPLC separation on an RP-18 column [MeOH/H_2O (8:2), Luna C18, 250 × 10 mm, 10 µm; λ = 280 nm], thus affording a fraction (t_R = 10 min) containing compound **1**. The fraction was then subjected to normal-phase HPLC on an SiO_2 column [*n*-Hex/*iso*-PrOH (8:2), Luna Silica, 250 × 4.6 mm, 5 µm; λ = 250 nm], which gave 15 µg of pure compound **1**.

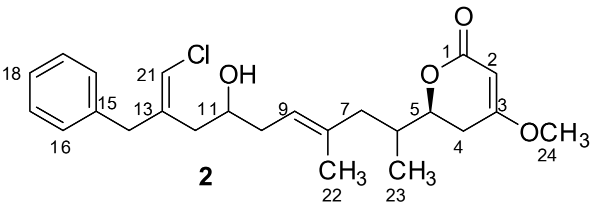

Smenolactone D (**2**)

Figure S4.Smenolactone D (2): colorless oil; UV (ACN): λ_{max} (ε) 232 (13500); ECD (ACN): λ_{max} (Δε) 248 (−12.8); HRESIMS (positive ion mode, MeOH) *m/z* 419.1980, $[M+H]^+$ (calcd for $C_{24}H_{32}ClO_4^+$, 419.1984, Δ −1.0 ppm); ^{1}H and ^{13}C NMR (CD_3OD)Table S2.

Pos.	δC, type	δH, mult (J in Hz)	HMBC a
1	170.9 (C)	-	
2	90.2 (CH)	5.19 (d, 1.6)	1,3,4
3	176.5 (C)	-	
4a	31.3 (CH2)	2.68 (ddd, 17.2, 13.2, 1.7)	2,3,4,5
4b		2.30 (br. dd (17.2, 3.5)	2,4
5	80.4 (CH)	4.35 (ddd, 13.1, 3.7, 3.7)	
6	35.4 (CH)	1.93 (m)	
7a	44.0 (CH2)	2.25 (m)	6,8,9,23
7b		1.93 (m)	6,8,9
8	136.2 (C)		
9	124.3 (CH)	5.25 (br. t, 7.0)	7,8,10,22
10a	37.2 (CH2)	2.19 (m)	8,9,11,12
10b		2.19 (m)	
11	71.1 (CH)	3.84 (m)	
12a	38.1 (CH2)	2.33 (m)	11,13,14,21
12b		2.22 (m)	11,13,14,21
13	142.1 (C)		
14	42.6 (CH2)	3.48 (br. s)	12,13,15,16/20,21
15	140.0 (C)		

International Journal of
Molecular Sciences

16/20	130.1 (CH)	7.18 (br. d, 7.6)	14,18
17/19	129.5 (CH)	7.29 (br. t, 7.6)	15,19/17
18	127.7 (CH)	7.20 (br. t, 7.6)	
21	116.2 (CH)	6.04 (br. s)	12,13,14
22	16.1 (CH3)	1.61 (br. s)	7,8,9
23	14.9 (CH3)	0.92 (d, 6.8)	5,6,7
24	57.4 (CH3)	3.80 (s)	3

[a] HMBC correlations from proton stated to the indicated carbon.

Table S2. NMR data for smenolactone D (**2**) (700 MHz, CD_3OD).

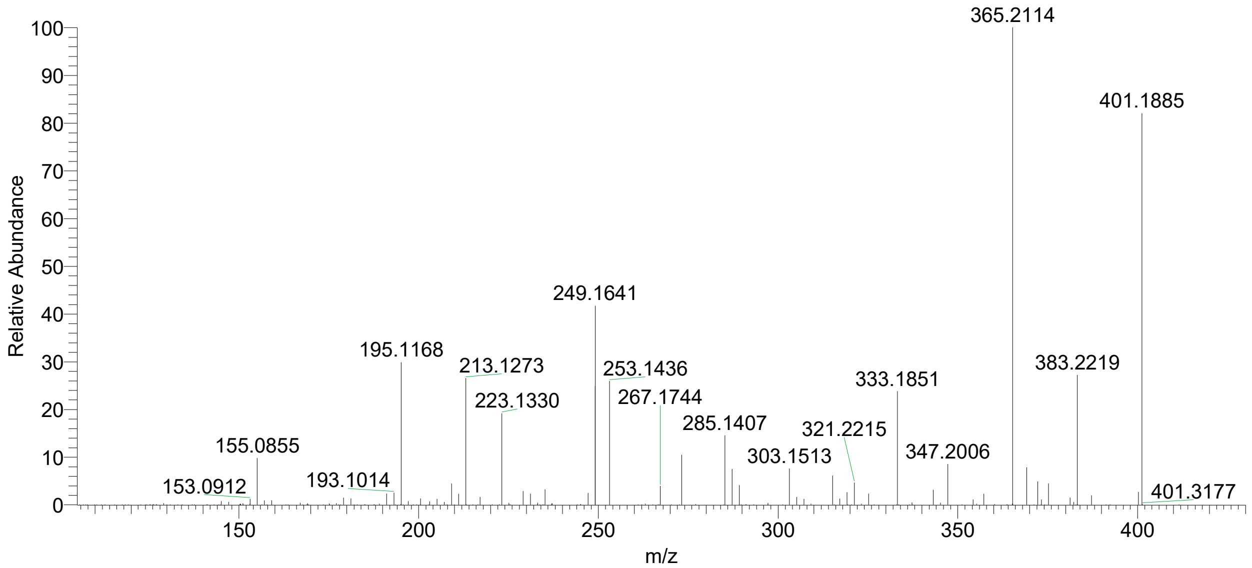

Figure S5. Positive ion mode high-resolution ESI MS/MS spectrum of smenolactone D (**2**), parent ion at *m*/*z* 419.19.

High performance liquid chromatography (HPLC) separations were achieved on an Agilent 1260 Infinity Quaternary LC apparatus equipped with a Diode-Array Detector (DAD).

The organic layer, containing smenamides and smenolactones, was subjected to reversed-phase HPLC separation [column 250 × 10 mm, 10 μm, Luna (Phenomenex) C18; eluent A: H_2O; eluent B: MeOH; gradient: 55→100% B, over 60 min, flow rate 5 $mLmin^{-1}$], thus affording a fraction containing compounds **2** that was purified on reversed-phase HPLC [column 250 × 4.6 mm, 5 μm, Luna (Phenomenex) C18; eluent A: H_2O; eluent B: ACN; gradient: 50→100% B, over 35 min, flow rate 1 mL min^{-1}], giving smenolactone D (**2**) (93 μg, t_R = 18.2 min).
